# An Endowment Effect Study in the European Union Emission Trading Market based on Trading Price and Price Fluctuation

**DOI:** 10.3390/ijerph17093343

**Published:** 2020-05-11

**Authors:** Jiqiang Wang, Fu Gu, Yingpeng Liu, Ying Fan, Jianfeng Guo

**Affiliations:** 1 College of Management and Economics, Tianjin University, Tianjin 300072, China; 13260439597@163.com; 2 Department of Industrial and System Engineering, Zhejiang University, Hangzhou 310027, China; gufu@zju.edu.cn; 3 Institutes of Science and Development, Chinese Academy of Sciences, Beijing 100190, China; liuyinpeng@casisd.cn; 4 School of Economics & Management, Beihang University, Beijing 100191, China; yfan1123@buaa.edu.cn

**Keywords:** EU ETS, endowment effect, WTA, WTP

## Abstract

This paper pioneers to investigate the endowment effect in the European Union mission Trading Scheme (EU ETS) as well as the impacts of trading experience and compliance pressure on the endowment effect. This study is based on the complete transaction records of the market. In the data set, the records of two consecutive reverse transactions from a same emitting company are selected. The lowest price that the buyer is willing to pay (WTP) and the maximum price the seller is willing to accept (WTA) are evaluated by excluding their risk cost that is used to avoid short-term fluctuations in the price. By distinguishing the difference between WTA and WTP, and long-term fluctuations in the prices during the two transactions, the trader’s endowment effect can be quantitively assessed. The results show that the degree of endowment effect of traders follows the trading experience. In addition, since the EU ETS is a cap-and-trade market, the traders face different levels of compliance pressure; when the pressure of the emission companies increases, the degree of endowment effect will also decrease.

## 1. Introduction

The endowment effect is a phenomenon: an agent has an increased preference towards an item, triggered by mere possession of that item [1]. In the case of trading, the endowment effect predicts an asymmetry between willing to pay (WTP) and willing to accept (WTA). The presence of endowment effect has been confirmed by various experiments [2,3]. Knetsch and Sinden [4] found the proof that the endowment effect is presented in parts of goods. Kahneman et al. [5] and List [6,7] showed that the endowment effect pervasively exists in consumer goods, but not in speculative goods. Yet, there is still controversy about the research process and explanation [8,9]. Recently, efforts are continuously been paid to identify the presence of the endowment effect in trading [10,11,12]. Moreover, researchers have reached an agreement: during the experiment, the behaviors of the experimenters may distort participants’ valuation, and thereby affect the participants’ decisions.

In the endowment effect literature, Knetsch and Sinden [4] opened up another hot topic, that is, the relation between the trading experience and the endowment effect. Kahneman et al. [5] designed the experiments to analyze this particular relation and found that trading experience does not weaken the endowment effect. However, the experimental study [2,3] might not be suitable for the accumulation of trading experience, which usually requires a long period. To overcome this shortcoming, List [6,7] selected experimental objects from the groups with and without trading experience. The results show that there is no noticeable endowment effect presented in the professional traders or consumers with trading experience, while the ordinary consumers and consumers without trading experience exhibit a significant endowment effect. Later, Horowitz and Mcconnell [13], and Tuncel and Hammitt [14] reviewed the extant literature and found a similar conclusion: the half of the studies support the existence of the endowment effect, while the other half disprove it. It is a constant debate on this topic [15,16], and no consensus has yet been reached. Regarding the origin of the controversy, Fehr et al. [10] and Bartling et al. [11] argued that all these studies were conducted in laboratories and the designs of the experiments had affected the experimenters; therefore, the results from the different experiments would hardly be consistent.

To address the above issue, this paper conducts an empirical analysis on the presence of the endowment effect in the European Union mission Trading Scheme (EU ETS). In addition, the EU ETS is a cap-and-trade market where its participants face compliance pressure of different levels and directions (i.e., compliance selling pressure and compliance buying pressure). The influences of compliance pressure on the endowment effect could affect the market liquidity of the EU ETS, and therefore this analysis could provide some implications for the development of the EU ETS. First, the market liquidity can be improved from the perspective of endowment effect, provided that the effect of the compliance pressure on the endowment effect is known. Second, when firms are buying (or selling) allowances, they are facing the corresponding compliance buying pressure (or the compliance selling pressure). By comparing the endowment effects on traders when they face performance pressures in different directions, policy suggestions can be provided from supply and demand aspect, and it will be helpful to promote the development of the market.

The selection of the EU ETS as the research object is based on the two following reasons. First, reviewing 76 endowment effects studies, Tuncel and Hammitt [14] found that the environmental field shows the highest level of the endowment effect. Besides, they suggested that when the uncertainty of the reference price is greater, the gap between WTA-WTP is wider [14], so the EU ETS which is characterized by violent price fluctuations can be a good choice for the endowment effect research. Second, in terms of theoretical origin, the endowment effect is an emerging branch of the behavioral finance theory, which has already been incorporated into the empirical study on the EU ETS, for examples, herd effect [17,18], price barriers [19], and price overreaction to information [17]. However, due to the limitations of the EU ETS database, most of the previous behavioral finance literature is only based on allowance price data. We have grasped the complete transaction data from 2008 to 2012 for our analysis; The amount of transactions is over more than one million, providing sufficient authenticity and accuracy of this analysis on endowment effect. In summary, the EU ETS is a suitable testing ground for behavioral finance research as well as endowment effect.

The contribution of this work is threefold. First, the endowment effects of the participants in the EU ETS is quantitively assessed. Based on the transaction prices and fluctuations of carbon prices, this study pioneers to evaluate and compares the WTP and WTA of traders in a real market, i.e., the EU ETS. Second, this work empirically analyzes how the degree of traders’ endowment effects changes with varieties in trading experience, compensating the extant literature which is filled with laboratory experiments. Third, based on the cap-and-trade attribute of the EU ETS, this paper includes the compliance pressure as an influential factor of the endowment effects, and conducts an empirical analysis of the impact of compliance pressure on the endowment effect. Considering the fact that low market liquidity is a problem that has plagued the EU ETS [20] and other carbon markets such as China [21], this study has practical significance for both policy makers and industrial practitioners in the ETS.

The structure of this article is as follows: the second part is the framework of the article; the third part is the introduction of transaction data in EU ETS, and the quantitative modeling for endowment effects, and the empirical model for the analyzes of the influences of trading experience and compliance pressure on the endowment effects; the fourth part is the analysis of the models’ results; the fifth part gives the conclusions and policy implications of this article; the last part points out the limitations of this research and potential future works.

## 2. Research Framework

**Figure 1:**
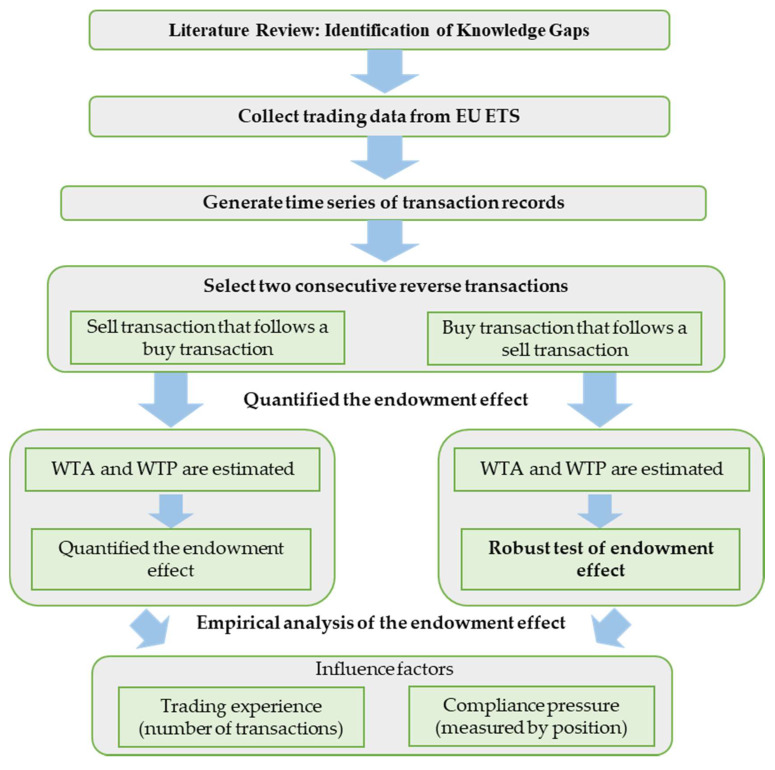
Research framework.

Based on the identified knowledge gaps, we conduct the research framework (see Figure 1). First, data of the EU ETS is collected and cleaned, and the complete transaction data is obtained. In this database, each emission company is taken as an individual, and the individual’s transaction records from 2008 to 2012 are formed into a time series. In each series, the records of two consecutive reverse transactions of the emitting enterprises are selected. Therefore, two types of the records are included: the first one is a selling transaction follows a buying transaction, and the second one is a buying transaction follows a selling transaction. The first type of records are used for analyzes of the endowment effect; based on the trading price, the trader’s WTA and WTP are evaluated by culling the risk cost paid by emitting companies to avoid short-term fluctuations in the price, and the risk cost is derived using the Value at Risk (VaR) model. After the differentiated value between WTA and WTP is obtained, the effect of long-term price fluctuations during the two trading periods is obtained through the Empirical Mode Decomposition (EMD) model, then the endowment effect of the trader is quantified accordingly. As to make the study more comprehensive, we can perform a robustness test on the endowment effect from another angle based on the second type of records mentioned above. If an individual buys allowance, whether the WTP in this transaction is much smaller than the WTA when it conducted the previous transaction. The process of the robustness test is identical to the above quantification process.

After quantifying the endowment effect of a trader, we conducted an empirical analysis of the effects of trading experience and compliance pressure on the endowment effect. Each emitting company’s trading experience is represented by the accumulated number of transactions before the current transaction, and the start time of the measurement for the number of transactions is May 1st, 2008, i.e., the beginning of the Phase II of EU ETS. The compliance pressure of each company is calculated from the gap of allocation and surrender and the trading volume before the current transaction. Similarly, in order to make the research more comprehensive, this article also conducts an empirical analysis on the endowment effect that is shown in the process of buying allowances after selling allowances, hence the influences of trading experience and compliance pressure on the buying behaviors that follows selling behaviors are analyzed.

## 3. Data and Methodology

### 3.1. Data

To achieve the 8% CO_2_ reduction commitment in the Kyoto Protocol, the EU ETS was launched in 2005. The EU ETS is the oldest and the currently largest emission allowance trading system in the world, operating as a market-based scheme with the purpose to reduce of carbon emissions.

**Figure 2:**
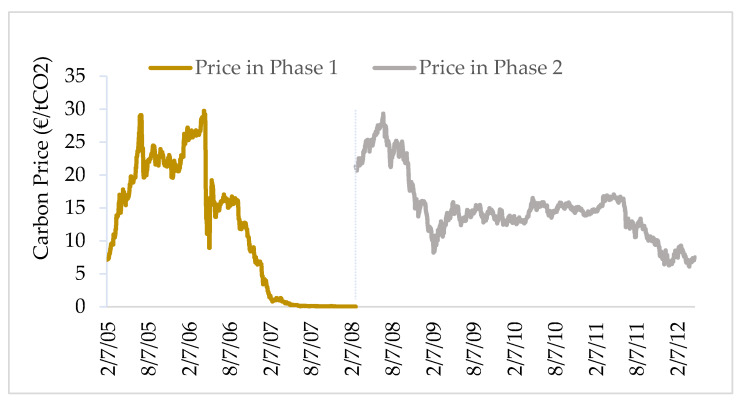
Trend of carbon spot prices in Phase I and Phase II. Source: ICE and BlueNext.

The EU ETS has been running for three phases. The first phase (the trial phase) was from 2005 to 2007; the second phase was from 2008 to 2012; and the third phase is from 2013 to 2020. Up to now, the first two phases of the EU ETS have been completed. Due to the over-allocation of allowances during the trial period, the carbon price has approximately dropped to 0 euros by the end of the first period (Figure 2), affecting the efficiency of the market transactions. In Phase II, the efficiency of the market is improved. Although the carbon price experienced a great decline during 2008 to 2009, the price was remained within the effective range. The European Union Transaction Log (EUTL) contains the trading records of all accounts in the EU ETS, and this work employs 361,432 transaction records taken place during the Phase II of the EU ETS for this empirical research.

During the Phase II, if a company makes two consecutive direction transactions, the records of transactions are selected. Therefore, two types of records are included: the first one is a selling transaction follows a buying transaction, and the second one is a buying transaction follows a selling transaction. According to this principle, a total of 65,077 transactions records are extracted from the full sample transaction records; 18,120 transactions records are selling follows buying, and 49,657 transactions records are buying follows selling.

### 3.2. Methodology

The endowment effect can be reflected in the gap between WTP and WTA. In a laboratory study of the endowment effect, the reference price of experiment goods is stable. While in the real market, the carbon price changes every day, accompanied by transaction risks. First, with the software of EViews, WTP and WTA are evaluated by excluding the risk cost that used to avoid short-term fluctuations in the trading price. The risk cost is acquisitioned from the VaR of carbon price, and the VaR is calculated based on the volatility item from the Autoregressive Moving Average-Exponential Generalized Autoregressive Conditional Heteroscedasticity (ARMA-EGARCH) model; secondly, the records of the two adjacent transactions by each trader are selected (i.e., selling allowances after buying allowances, or buying allowances after selling allowances). After the difference value between WTA and WTP is obtained, this study subtracts the effect of the long-term price fluctuations during the two transactions, then the endowment effect of the trader is quantified accordingly. The long-term price fluctuations are calculated through constructing an EMD method on Python 3(Guido van Rossum; open-source )finally, the impacts of trading experience and compliance pressure on the endowment effect can be analyzed through EViews 10 9 (IHS Global INC., Irvine, CA, USA).

#### 3.2.1. ARMA-EGARCH Model for Carbon Price

To generalize the implausible assumption of a constant variance should be existed in a studied period, a stochastic process called Autoregressive conditional heteroskedasticity (ARCH) processes were introduced by Engle et al. [22]. The ARCH model is appropriate when the error variance in a time series follows an AR model, if an ARMA model is assumed for the error variance, the model can be a GARCH model [23]. 

To explain the trend and volatility of carbon prices in Phase I, Fan et al. [24] constructed an AR-GARCH model and found that the pattern of the carbon prices was differentiated from that of Phase I (Figure 2). Hence, we construct an ARMA-EGARCH model to investigate the carbon prices in Phase II. The item of MA represents deviation of the actual price and forecast price in the past days, as the market was more mature and can make self-adjustments in Phase II [25]. To ensure the smoothness of the price’s data, the returns of carbon price are calculated in Equation (1):

(1) $${\mathrm{RCP}}_{t}{=\mathrm{lnP}}_{t}{\;-\;\mathrm{lnP}}_{t-1}$$

where ${\mathrm{RCP}}_{t}$ is the logarithm returns of the carbon price at time t, and $P_{t}$ is the carbon price at time t.

The mean equation of the ARMA-EGARCH model is shown as follows

(2) $${\mathrm{RCP}}_{t}{=\theta }_{1}{+\theta }_{2}\mathrm{AR}\left(1\right){+\theta }_{3}\mathrm{MA}\left(1\right){+\varepsilon }_{t}$$

where $\theta_{1}$ is the constant, $\theta_{2}$ and $\theta_{3}$ are the coefficients of AR and MA, respectively, the variance equation is shown as follows:

(3) $${\log\sigma }_{t}^{2}{=\beta }_{1}{+\beta }_{2}{\log\delta }_{t-1}^{2}{+\beta }_{3}\left|\frac{\varepsilon_{t-1}}{\delta_{t-1}}\right|{+\beta }_{4}\frac{\varepsilon_{t-1}}{\delta_{t-1}}$$

where $\beta_{1}$ measures the degree of volatility persistence for ‘carbon pricing’, and $\varepsilon_{t-1}$/$\sigma_{t-1}$ represents the standardized innovations. If $\beta_{3}$ is not equal to zero, an asymmetry effect is uncovered.

#### 3.2.2. Empirical Mode Decomposition (EMD) Model for Carbon Price

The conventional signal processing techniques, including time-domain and frequency-domain analysis, are based on the assumption that the process generating signals is stationary and linear, as both of them depend on the Fourier transform. Huang et al. [26] proposed a time–frequency analysis method, i.e., the EMD method, for nonlinear and nonstationary signals. The basic principle of EMD is to smooth the signal and to decompose the fluctuations of different scales. Therefore, a series of subsequences with different frequencies and amplitudes and a trend term can be obtained, and each subsequence represents the original sequence at different scales and corresponds to an intrinsic mode functions (IMF). The basic algorithm of the EMD algorithm uses the average value of the upper and lower envelopes to determine the “instantaneous equilibrium position”, and then extract the IMF. 

The EMD method is an adaptive and powerful time–frequency signal processing technique. The EMD technique is used as a tool for extracting the required information from a noisy signal [27,28] and it has been implemented for condition monitoring of rotating machinery such as bearing, gears, rotors, and denoising of signals [29]. In this study, the EMD method is used in the decomposition of carbon price, and the specific steps of the EMD decomposition are as follows:

Step 1. Identify all local maxima and minima in $P_{t}$, and use cubic spline interpolation to fit the upper and lower envelopes of $P_{t}$. Then use $m_{t}$ to represent the mean value of the upper and lower envelopes at time t.

Step 2. Equation (4) calculates a new sequence $d_{t}$.

(4) $$d_{t}{=p}_{t}{\;-\;m}_{t}$$

Then, whether $d_{t}$ is an IMF is determined according to the value of $S_{d}$ shown in Equation (5).

(5) $$S_{d}=\frac{\sum_{t=0}^{T}{|d}_{i,t}\;{-\;d}_{i-1,t}{|}^{2}}{\sum_{t=o}^{T}d_{i,t}^{2}}$$

where $d_{i,t}$ is the result of the i times screening, and the threshold of $S_{d}$ is usually set between 0.2 and 0.3. If the value of $S_{d}$ is smaller than the threshold, the screening process is stopped; otherwise, $d_{t}$ is regarded as a new sequence $P_{t}$ to be decomposed, and the above iterative process is performed again.

Step. 3. If $d_{t}$ satisfies the stopping condition of the above screening process, then $d_{t}$ is an IMF, and $d_{t}$ will be separated from $P_{t}$ to obtain the remainder $r_{t}$

(6) $$r_{t}{=p}_{t}{\;-\;d}_{t}$$

Step 4. If the remaining term $r_{t}$ has become a monotonic function or constant, or the amplitude is lower than the predetermined threshold value, which will result in that the IMF cannot be further extracted, the entire decomposition process ends; otherwise, $r_{t}$ is regarded as a new sequence $P_{t}$ to be decomposed, return to Step 1, and re-execute the above iterative process.

When the decomposition is completed, the initial sequence of carbon price $P_{t}$ is iteratively decomposed into orthogonal IMFs represented by $c_{i,t}$ and trend residuals $R_{n,t}$, i = 1, 2, ... , n, as shown in Equation (4), where $c_{i,t}$ takes the $d_{t}$ obtained in Step 3 in order.

(7) $$P_{t}=\overset{n}{\underset{i=1}{\sum }}c_{i,t}{+R}_{n,t}$$

#### 3.2.3. Quantify the Endowment Effect

In the laboratory research on the endowment effects, WTA and WTP can be known directly by asking individuals [5,6]. Typically, it is difficult to access the WTA and WTP of the individuals in the real trading market, because the trading price is not exactly equivalent to the WTA and WTP. In the experiment, the reference price of the traded item will not change, no matter whether it is a cola or a pen. However, due to the changes in supply and demand, the carbon prices in the EU ETS vary daily (Figure 2). Violent price fluctuations are always accompanied with trading risks; when traders buy allowances (sell allowances), they will face market risks of price declines (price increases). It is assumed that traders are risk averter, and they are willing to pay a risk cost to avoid the risk of price fluctuations. Therefore, the actual trading price of the trader is formed by the WTP (WTA) plus the risk cost paid to avoid short-term price fluctuation. In this study, the risk cost is estimated with the inference of VaR, and it is assumed that the risk cost is equal to the absolute vale of VaR.

In 1993, the member states of G30 published a report on financial derivatives, suggesting the use of VaR to assess financial risk for the first time. In the financial industry, VaR is the standard measure of risk and has been popular since it was applied in Basel II (Basel Committee, 1996) as the benchmark for regulatory capital. VaR has been extensively conducted in empirical analysis, and is widely used by investors, investment banks, commercial banks, and market regulators. The current mainstream calculation method of VaR is based on the ARCH models. This is mainly because ARCH models have a very good performance in the field of describing the volatility of financial market asset returns, and thus VaR value can more clearly reflect financial risk. VaR represents the maximum possible loss of a certain portfolio under normal market conditions at a given confidence level and holding period. 

**Figure 3:**
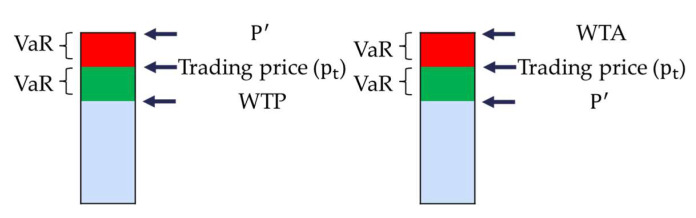
Explanation on the relationship among Value at Risk (Var), trading price and WTP (WTA).

In each compliance year, if emitting companies are not allocated with sufficient allowances to surrender, they would buy allowances to avoid penalties; if emitting companies have surpluses on their allowances, they can sell some allowances to realize the profits from emission reductions. It is assumed that every company is pursuing cost minimization or maximizing profits. ${\mathrm{VaR}}_{t}$ is used to represent the fluctuation range of the carbon price. The reason for emitting companies chose to buy allowances at time t is they believe the price will rise to *P’* soon, therefore they are willing to pay risk cost (green fraction in Figure 3 left) to avoid the price rising to *P’*. Thus, the WTP is calculated by minus the value of green fraction (Figure 3 left) from the trading price. Similarly, the reason for emitting companies chose to sell allowances at time t is that they believe the price will decrease to P’ soon, so they are willing to pay some risk cost (red fraction in Figure 3 right) to avoid the price decreasing to *P’*, therefore the WTA is calculated by plus the value of red fraction (Figure 3 right) on the trading price.

In this study, $R_{t}$ is the daily series of the returns of the carbon price, and *z (α)* is the quantile corresponding to the probability level *α* in the standard normal distribution. In the 5% significance level, the value is 1.645. $\delta_{t}$ is the conditional standard deviation series of the GARCH Equation (7), and $P_{t}$ is the original carbon price. ${\mathrm{VaR}}_{t}$ is calculated as follows:

(8) $${\mathrm{VaR}}_{t}{=(R}_{t\;}\;\pm \;1{.645\delta }_{t}{)P}_{t}$$

where the plus sign in parentheses represents the risk of price increase, and the minus sign represents the risk of price decrease.

When a company needs to buy allowances, it is facing the risk of prices rising in the next moment, and the ${\mathrm{VaR}}_{t}$ is shown as below:

(9) $${\mathrm{VaR}}_{t}{=(R}_{t\;}+1{.645\delta }_{t}{)P}_{t}$$

The actual trading price is the WTP of the trader plus the risk cost (VaR) paid to avoid the risk of prices rising, therefore the calculation method for WTP is shown as follows:

(10) $${\mathrm{WTP}=P}_{t}\;{-\;\mathrm{VaR}}_{t}$$

When a company needs to purchase allowances, it is facing the risk of prices decrease in the next moment, and the ${\mathrm{VaR}}_{t}$ is shown as below:

(11) $${\mathrm{VaR}}_{t}{=(R}_{t\;}\;-\;1{.645\delta }_{t}{)P}_{t}$$

The actual trading price is the WTA of the trader minus the risk cost (VaR) paid to avoid the risk of prices rising. Since the risk cost is a positive, we use (−VaR) to represent that value, and the calculation method of the WTA is shown as follows:

(12) $${\mathrm{WTA}=P}_{t}{+(-\mathrm{VaR}}_{t})$$

First of all, endowment effect analysis is performed on the first type of data, that is, transaction data of selling allowances that follows buying allowances. Next, compare the WTA for selling allowances and the WTP for buying allowances, and then remove the effect of long-term fluctuations in carbon prices between two trading periods, namely, the difference between the trend terms of the EMD model at the two trading times. Finally, the endowment effect is quantified:

(13) $${\mathrm{EE}=\mathrm{WTA}\;-\;\mathrm{WTP}\;-\;(\mathrm{TP}}_{s}\;{-\;\mathrm{TP}}_{b})$$

where EE is the quantified endowment effect, ${\mathrm{TP}}_{s}$ is the trend of price when the trader conducted selling transaction, and ${\mathrm{TP}}_{b}$ is the trend of price when the trader conducted buying transaction.

Due to the comprehensiveness of the used data set, we can perform a robustness test on the endowment effect from another perspective. In the test, the second type of data is selected, that is, the transaction data of buying allowances that follows selling allowances. The robustness test is achieved through analyzing whether the WTP of the buying allowances is much lower than the WTA of selling allowances, and the Equation (14) of robust test is similar to Equation (13):

(14) $${\mathrm{RTEE}=\mathrm{WTP}\;-\;\mathrm{WTA}\;-\;(\mathrm{TP}}_{b}{\;-\;\mathrm{TP}}_{s})$$

where RTEE is the robust test of endowment effect, ${\mathrm{TP}}_{b}$ is the trend of price when the trader made buying transaction, and ${\mathrm{TP}}_{s}$ is the trend of price when the trader made selling transaction.

#### 3.2.4. Model the Influences of Trading Experience and Compliance Pressure on the Endowment Effect

Because the market is a cap-and-trade market, different emitting companies are allocated with unequal allowances in each year and would surrender allowances that are equal to their emissions, therefore different companies could have different gaps between allocation and surrender. If emitting companies have surpluses in allowances, they need to sell those to benefit from carbon reduction; if they do not have enough allowances to surrender, they need to buy allowances to avoid the penalty. Therefore, different companies have different compliance pressure, which is reflected by the gap, this paper also models and analyzes the impact of compliance pressure on the endowment effects.

When the traders conducted trading at time t, their trading experience are represented by the accumulated number of transactions before time t, and the start time of measurement for the number of transactions is May 1st, 2008, i.e., the beginning of Phase II of the EU ETS. Traders’ compliance pressure is reflected in their positions, and their initial position is defined as the subtraction value between allocation and surrender in the first year of a phase. When the company makes transactions, the position is adjusted according the trading volume. When the company is allocated allowances for another year, the position of the company will be subjoined the subtraction value of allocation and surrender for the second year. Hence, the empirical research about the effects of trading experience and compliance pressure on endowment effects is conducted:

(15) $${\mathrm{EE}}_{i,t}{=c}_{1}{+\beta }_{1,1}{\mathrm{TE}}_{i,t}{+\beta }_{1,2}{\mathrm{POS}}_{i,t}$$

where ${\mathrm{EE}}_{i,t}$ is the quantified endowment effect of emitting company i at time t, ${\mathrm{TE}}_{i,t}$ represents the trading experiences of the company i at time t, and ${\mathrm{POS}}_{i,t}$ represents the compliance pressure of the company i at time t.

In the robustness test, we also analyze the endowment effect reflected in the second type of transactions, i.e., a buying transaction that follows a selling transaction. The effects of the trader’s trading experience and compliance pressure on the endowment effect are as follows:

(16) $${\mathrm{RTEE}}_{i,t}{=c}_{2}{+\beta }_{2,1}{\mathrm{TE}}_{i,t}{+\beta }_{2,2}{(-\mathrm{POS}}_{i,t})$$

where ${\mathrm{RTEE}\;}_{i,\;t}$ is the absolute value of that calculated from Equation (14), and it represents the quantified endowment effect of emitting company i at time t. To facilitate the analysis on the results, we take the inverse of ${\mathrm{POS}}_{i,t}$, as most traders had a negative position when they conducted buying transaction in the EU ETS.

## 4. Results

### 4.1. ARMA-EGARCH Results

The results of the ARMA-EGARCH model for the ‘carbon pricing’ are summarized in Table 1. Notably, most of the coefficients are significant at 1% level, except for $\beta_{3}$ and $\beta_{4}$, which are significant at 5% level. This indicates that the models (Equation (3) and Equation (4)) are rational and valid. In the ARMA-EGARCH model, $\theta_{1}$ and $\beta_{1}$ are constants. According to the significant values of $\theta_{2}$ −0.894) and $\theta_{3}$ (0.926), the carbon price in t depends significantly on its own lagged values and the deviation of the actual price and forecast price in t−1. 

The volatility persistence of the ‘compliance trading’ power is measured by the parameter $\beta_{2}$ (0.438), which is significant, showing strong volatility persistence. $\beta_{3}$ (0.048) and $\beta_{4}$ (−0.135) measure the interpretation of standardization shocks on conditional Variance, the asymmetry of volatility can be confirmed by these two significant values.

### 4.2. EMD Results

**Figure 4:**
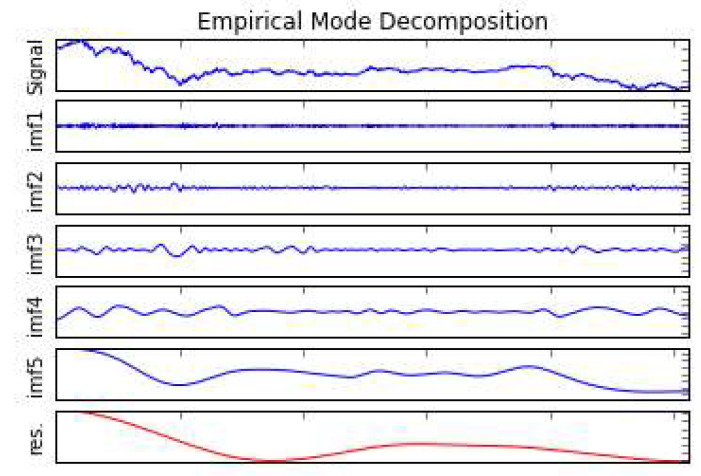
Results of the Empirical Mode Decomposition.

The EMD decomposes the original signal data into several clear, periodic IMF components. In economics, each component can be analyzed and explained according to different influencing factors to find the development law. According to the adaptability of the EMD method, this paper directly performs EMD decomposition on the original carbon price and obtains 5 IMF components and 1 residual term. The decomposition results are shown in Figure 4, the IMF components of which are arranged in descending order of frequency and the last residual term is the trend amount. The IMFs respectively show the different frequencies and amplitudes of the carbon prices in the EU carbon market throughout the research period. The focus of this analysis is the trend item by excluding different fluctuations from original carbon price. From the trend in Figure 4, we can roughly see that from May 2008 to mid-2009, the carbon price showed a downward trend. During the following period, it shows an upward trend which remained at its peak until the end of 2010, and then continued to decline until April 2012.

### 4.3. Quantitively Assessed Results of Endowment Effect

From the complete transaction data, there are 17,895 records of selling allowances that follows buying allowances, and 46,437 records of buying allowances that follows selling allowances. Calculated by Equation (13), 15,617 records show positive values, accounting for 87% of the total selling transactions that follows buying transactions and indicating the existence of the endowment effect. Calculated by Equation (14), 41,186 records show negative values, accounting for 89% of the total buying transactions that follows selling transactions and further verifies the existence of the endowment effect. Moreover, the 15,617 selling transactions are conducted by 2296 traders, and 41,186 buying transactions are conducted by 2702 companies. Amongst them, there are 1956 traders can be found in both the categories, indicating that the companies have stable performances on the endowment effect, no matter whether they are selling or buying allowances.

### 4.4. The Influences of Trading Experience and Compliance Pressure on the Endowment Effect 

The results of the model for the ‘endowment effect’ are summarized in Table 2. Obviously, all of the coefficients are significant at the 1% level, which indicates that the models (Equation (15) and Equation (16)) are rational and valid, and trading experience and compliance pressure can affect the endowment effect. Both the values of $\beta_{1,1}$ (−2.22 × 10^−5^) and $\beta_{1,2}$ (−5.33 × 10^−9^) are negative, indicating that the trading experience and compliance pressure can weaken the endowment effect, and this is in line with the findings of List (2003). As the endowment is quantified through the difference between WTA and WTP, and WTP is an expected price of the trader in the previous transaction, the trading experience and compliance pressure can lower the WTA of trader. In the Equation (16), $\beta_{2,1}$ (−3.47 × 10^−6^) and $\beta_{2,2}$ (−7.05 × 10^−9^) are also the counter evidence to the above findings. The difference is that the trading experience and compliance pressure can higher the WTP of trader, and then weaken the endowment effect.

By comparing the absolute values of the model coefficients $\beta_{1,1}$ and $\beta_{1,2}$ (or $\beta_{2,1}$ and $\beta_{2,2}$), it can be roughly seen that in EU ETS, the effect of trading experience on the endowment effect is greater than that of compliance pressure. The absolute value of $\beta_{1,1}$ is much larger than that of $\beta_{2,1}$, showing that the effect of the trading experience on WTA of selling allowances is greater than that on WTP of buying allowances. This is because the trading purposes are different. In the EU ETS, most allowances are allocated for free, therefore companies may make profits from selling allowances, no matter what the carbon price is. However, companies have always sought to minimize costs when they buy allowances, and therefore trading experience have less impacts on buying allowances. The absolute value of $\beta_{1,2}$ is much smaller than the absolute value of $\beta_{2,2}$, which shows that the effect of trading experience on WTP of buying allowances is greater than that on WTA of selling allowances. In Phase II of the EU ETS, emitting companies are allocated allowances at the beginning of each year and surrender allowances at the end of the year. If there are remaining allowances, they can be used for the next year. However, if the allowances are not enough for surrender, the emitting companies would face fines, and thus the compliance pressure has stronger influences on buying allowances.

## 5. Conclusions

In this paper, for the first time, the presence of the endowment effect is analyzed in a real market (the EU ETS) based on the complete transaction data from 2008 to 2012, and whether the endowment effect will be influenced by trading experience is also investigated. In addition, in the cap-and-trade market mechanism, this article studies the impact of compliance pressure on the endowment effect in the EU ETS.

This article has two findings in the field of economics. First, the endowment effect is observed and quantified in a real market based on the transaction data, once companies buy carbon allowances, they have a higher valuation of the carbon price. Contrarily, once companies sell carbon allowances, they have a lower valuation of the carbon price; second, in previous laboratory research, due to lack of sufficient experiment time, it is still controversial whether trading experience will affect the endowment effect. This paper conducts an empirical analysis based on 361,432 records of individuals transaction, and it lasted for five years (from 2008 to 2012). It is found that the companies have the endowment effect regardless of trading experience, but when the trading experience increases, the endowment effect will be weakened.

This article also has two findings in the field of carbon market. First, this article finds that in this cap-and-trade market, the traders are not only chasing for achieving compliance obligation, but also are seeking to make profits through transactions. When traders conduct selling transaction follows a buying transaction, the WTA of selling is usually greater than the WTP of buying. Similarly, when traders conduct a buying transaction follows a selling transaction, the WTP of buying is often lower than the WTA of selling. Second, the companies’ endowment effect is influenced by the compliance pressure in the EU ETS; the higher compliance pressure they face, the lower level of the endowment effect they perform. Moreover, compared with selling allowances, compliance pressure has stronger influences on buying allowances, and increase their willingness to buy. This is consistent with the finding of Kahneman et al. [5]: the inability to trade is due to the endowment effect, and it is mainly owning to the seller’s unwillingness to sell.

Based on the findings, three policy implications that aim at improving the effectiveness of the carbon market can be offered. First, this article finds that endowment effects are pervasively existed in the EU ETS, which is reflected in the gap between WTP and WTA and can affect the liquidity of allowances. While the trading experience can reduce the magnitude of the endowment effects, therefore, market managers can encourage traders to conduct allowance transactions of small volumes, through which traders can accumulate trading experience, thereby weakening the endowment effect and promoting the market liquidity.

Second, in this particular cap-and-trade market, the companies generally face varying degrees of compliance pressure according to their volumes of allocation and emission. This study finds that compliance pressure can reduce endowment effects, therefore, the companies can be allocated a little more allowances than their emissions if they actively invest in reducing emissions, on the contrary, the companies can be allocated a little less allowances than their emissions if they do not actively invest in reducing emissions. This can not only encourage enterprises to invest in emission reduction technology, but also form compliance pressure on emitting companies in the EU ETS and to promote the conclusion of transactions.

Finally, this research shows that in the EU ETS, endowment effects make transactions difficult to reach, which is mainly owning to the seller’s unwillingness to sell. It means that apart from avoiding the situation of over-allocation in allowances, avoiding the circumstances that traders cannot find a counterparty to buy allowances is also important. According to this finding, when formulating the plan of emission reduction, it is necessary to set the volume of allocation a slightly larger than that of emission, ensuring that companies with shortages of allowances can buy enough allowances to promote smooth market operations

### Shortcomings and Future Work

Admittedly, there is still a shortcoming in this study. Since only the transaction price is recorded in the dataset of the EU ETS, it is impossible to directly obtain the WTA and WTP of traders in each transaction. Therefore, this article can only estimate the WTA and WTP by excluding the risk cost used to avoid short-term price fluctuations from the trading price; this estimation process is flawed. Nevertheless, this research is still a meaningful exploration of the endowment effect of traders in a real market, and it has certain reference significance for future research on endowment effect in the real market.

This empirical research is based on the of 361,432 full-sample transaction data of the EU carbon market. It is a preliminary study of the endowment effect in a real market. In potential future research, we will distinguish and quantify the risk appetites of the traders and make more accurate estimations of their WTA and WTP according to the risk appetite of traders, therefore we can quantify the endowment effect more accurately.

## Figures and Tables

**Table 1:** Estimated parameters of the ARMA-EGARCH model for carbon price.

Parameter	Coefficient	Std. Error	*z*-Statistic	Prob.
θ1	−5.88E-05	0.001	−0.106	0.916
θ2	−0.894	0.042	−21.388	0.000 ***
θ3	0.926	0.033	28.170	0.000 ***
β1	−8.955	0.436	−20.537	0.000 ***
β2	0.438	0.033	13.095	0.000 ***
β3	0.048	0.024	2.039	0.042 **
β4	−0.135	0.057	−2.353	0.019 **

Note: The AR (1)-EGARCH (1,1) model was selected based on Akaike Information Criterion (AIC). *** and ** denote significance at 1% and 5% levels, respectively.

**Table 2:** Estimated parameters of the model for the Endowment Effect.

Parameter	Coefficient	Std. Error	t-Statistic	Prob.
$C_{1}$	1.312	0.007	178.874	0.000 ***
$\beta_{1,1}$	−2.22 × 10^−5^	2.75 × 10^−6^	−8.085	0.000 ***
$\beta_{1,2}$	−5.33 × 10^−9^	1.03 × 10^−9^	−5.161	0.000 ***
$C_{2}$	−1.372	0.006	−245.969	0.000 ***
$\beta_{2,1}$	−3.47 × 10^−6^	4.30 × 10^−7^	−8.084	0.000 ***
$\beta_{2,2}$	−7.05 × 10^−9^	1.18 × 10^−9^	−5.998	0.000 ***

Note: *** and ** denote significance at 1% and 5% levels, respectively.
